# ﻿*Petrocosmea pengzhouensis* (Gesneriaceae), a new species from Sichuan Province, China

**DOI:** 10.3897/phytokeys.262.164617

**Published:** 2025-09-04

**Authors:** Xinyi Zheng, Tianmeng Qu, Xinyu Chen, Yanru Zhang, Jin Wang, Li Li, Wen Wu, Yujie Wang, Bo Li, Ke Huang, Zhixi Fu

**Affiliations:** 1 Key Laboratory of Land Resources Evaluation and Monitoring in Southwest, Sichuan Normal University, Ministry of Education, Chengdu 610066, Sichuan, China; 2 College of Life Sciences, Sichuan Normal University, Chengdu 610101, Sichuan, China; 3 Chengdu Mountain Culture Communication Co., Ltd., Chengdu 610014, Sichuan, China; 4 Chengdu Bird Watching Society, Sichuan Wildlife Conservation Association, Chengdu 610041, Sichuan, China; 5 Pengzhou Monitoring Station of Chengdu Pollution Source Monitoring Center, Chengdu 611930, Sichuan, China; 6 Chengdu Ecological and Environment Monitoring Center Station, Chengdu 610066, Sichuan, China; 7 Sichuan Ecological Environmental Monitoring Center, Chengdu 610091, Sichuan, China; 8 Sustainable Development Research Center of Resources and Environment of Western Sichuan, Sichuan Normal University, Chengdu 610066, Sichuan, China

**Keywords:** Morphological characters, new species, *
Petrocosmea
*, taxonomy

## Abstract

A new species of Gesneriaceae, *Petrocosmea pengzhouensis* K.Huang & Z.X.Fu, **sp. nov.**, from the Feilaifeng Scenic Area of Longmen Mountain National Geological Park (Pengzhou, Chengdu City, Sichuan Province, China), is described and illustrated. Morphological and phylogenetic analyses provide robust evidence for its recognition as a new species. While morphologically most similar to *P. duclouxii* Craib, phylogenetic reconstructions reveal its closest affinities with *P. duclouxii* and *P. intraglabra* (W. T. Wang) Z. J. Qiu. The new species is morphologically distinguished from *P. duclouxii* by a suite of diagnostic characters: leaf shape (ovate to oblique-ovate vs. ovate to nearly orbicular), corolla throat (spotless vs. bearing two deep purple spots), and filament pubescence (translucent-puberulent vs. rust-brown puberulent). A distribution map and a comparative morphological table with *P. duclouxii*, *P. intraglabra*, and *P. hexiensis* S. Z. Zhang & Z. Y. Liu, and a preliminary conservation assessment following IUCN criteria are provided.

## ﻿Introduction

*Petrocosmea* Oliv., a genus within the Gesneriaceae family and subfamily Didymocarpoideae (Weber et al. 2013; Qiu et al. 2020; Tang et al. 2022), has become an important indoor ornamental cultivar due to its emerald-green leaves, brightly colored flowers, and unique morphology (Kan et al. 2024). In 1985, Wang conducted a taxonomic revision of this genus, which was then considered to comprise 27 species and four varieties classified into three sections: sect. Anisochilus Hemsl., sect. Deinanthera W. T. Wang, and sect. Petrocosmea Oliv. (Wang 1985). In 2015, based on the first phylogenetic study, the genus was reclassified to include 33 species and three varieties distributed across five sections, with the addition of sect. Minor Zhi J. Qiu and sect. Barbata Zhi J. Qiu (Wang et al. 1990, 1998; Qiu et al. 2015; Tang et al. 2021).

To date, approximately 70 species of *Petrocosmea* have been reported worldwide (Li et al. 2023a; GRC 2025). Most *Petrocosmea* species exhibit narrow-range distributions (Wei and Wen 2009; Tang et al. 2021) and are primarily found in southern and southwestern China, which represent two major diversity centers of Gesneriaceae (Xu and Liu 2011; Tang et al. 2021). According to the Catalog of Life China 2025 edition, 11 *Petrocosmea* species are distributed in Sichuan Province, southwestern China (http://www.sp2000.org.cn/).

During field expeditions in Pengzhou, Chengdu City, Sichuan Province, in April 2025, we discovered living specimens of *Petrocosmea* (Gesneriaceae) growing on limestone cliffs within the Feilaifeng Scenic Area of Longmen Mountain National Geopark. Flowering specimens were collected for further study. These plants inhabit moist limestone cliff environments. Their rosette growth habit, cymes with solitary flowers, pale purple corollas, and anther dehiscence patterns show remarkable similarity to those of *P. duclouxii*, suggesting their possible taxonomic placement within sect. Petrocosmea. Based on preliminary morphological comparison and molecular evidence, we confirm that their comprehensive characteristics differ from those of previously reported taxa, thus supporting their recognition as a new species, which we formally describe here.

## ﻿Material and methods

### ﻿Sample collection and morphological analysis

In April 2025, living materials and voucher specimens of the new species were collected from limestone cliffs in Pengzhou, Chengdu City, Sichuan Province. The morphology of the new species was comprehensively compared with its closest relatives, *P. duclouxii* and *P. intraglabra*. Type specimens have been deposited in the herbarium of Sichuan Normal University (**SCNU**). The conservation status of the new species was assessed following the IUCN Red List Categories and Criteria (IUCN 2024).

### ﻿DNA extraction and sequencing

Total genomic DNA was extracted using the modified CTAB method (Doyle and Doyle 1987). Paired-end DNA libraries were prepared following the Illumina DNA Library Construction Protocol (Allen et al. 2006) and sequenced on an Illumina HiSeq X Ten platform (San Diego, CA, USA). High-quality reads were assembled using GetOrganelle v1.7.2 (Jin et al. 2020) with default parameters. After annotation analysis using CPGAVAS2, the results were manually checked and adjusted in Geneious (Kearse et al. 2012; Shi et al. 2019). The gene map of the plastomes was generated using the Organellar Genome Draw (OGDraw) tool (https://chlorobox.mpimp-golm.mpg.de/OGDraw.html, accessed on 6 June 2025) (Greiner et al. 2019). Various plastid features, such as gene length and GC content, were analyzed using CPJSdraw (Li et al. 2023b). The complete chloroplast genome sequence and ITS (internal transcribed spacer) sequence are deposited in the NCBI GenBank database (http://www.ncbi.nlm.nih.gov/) with registration numbers PV750053 and PV819303 (*P. pengzhouensis*), respectively.

### ﻿Phylogenetic analysis

Based on previous studies (Qiu et al. 2015), we obtained chloroplast DNA regions (*rps*16, *trn*T-L, etc.) and ITS sequences of 37 *Petrocosmea* species (including the newly discovered species) and two outgroup species (*Raphiocarpus begoniifolius* and *R. petelotii*) (Table 1). After comparing nodal support values and the topological structure of phylogenetic trees constructed separately or in combination with different chloroplast DNA fragments and ITS sequences, *rps16*, *trnT-L*, and ITS sequences were selected as the optimal molecular combinations in this study. Sequence alignments for each of the three selected markers were performed using MAFFT v7.520 (Katoh and Standley 2013) with default parameters. The aligned sequences were then trimmed with the “trimAL Wrapper” in TBtools v2.322 to remove poorly aligned regions. The processed sequences were concatenated using SequenceMatrix 1.10 to generate a combined dataset for phylogenetic reconstruction.

**Table 1.** Species and GenBank accession number for taxa included for phylogenetic reconstruction in this study.

	Taxon	*rps*16	*trn*T-L	ITS
**Ingroups**	*Petrocosmea pengzhouensis*	PV750053	PV750053	PV819303
	*P. barbata* Craib	KR006491	KR006424	KR006475
	*P. begoniifolia* C. Y. Wu & H. W. Li	KR006494	KR006431	KR006482
	*P. cavaleriei* H. Lévl.	KR006487	KR006420	KR006476
	*P. coerulea* C. Y. Wu & W. T. Wang	KR006492	KR006427	KR006483
	*P. duclouxii* Craib	KR006498	KR006433	KR006478
	*P. flaccida* Craib	KR006517	KR006414	KR006471
	*P. forrestii* Craib	KR006520	KR006416	KR006464
	*P. glabristoma* Z. J. Qiu & Y. Z. Wang.	KR006516	KR006417	KR006468
	*P. grandiflora* Hemsl.	KR006504	KR006437	KR006467
	*P. grandifolia* W. T. Wang	KR006507	JN092505	JN092439
	*P. hexiensis* S. Z. Zhang & Z. Y. Liu	KR006497	KR006415	KR006469
	*P. huangjiangensis* Yan Liu & W. B. Xu	KR006503	KR006435	KR006484
	*P. iodioides* Hemsl.	KR006513	JN092506	JN092440
	*P. kerrii* Craib	KR006509	JN092507	JN092441
	P. kerrii Craib var. crinita W. T. Wang	KR006505	KR006425	KR006462
	*P. longianthera* Z. J. Qiu & Y. Z. Wang	KR006514	JN092481	JN092398
	*P. longipedicellata* W.T.Wang	KR006490	KR006422	KR006474
	*P. mairei* H. Lévl.	KR006519	KR006418	KR006465
	*P. intraglabra* (W. T. Wang) Z. J. Qiu	KR006495	KR006430	KR006479
	*P. martinii* H. Lévl.	KR006501	JN092508	JN092442
	P. martinii var. leiandra W. T. Wang	KR006512	JN092509	JN092443
	*P. melanophthalma* Huan C. Wang, Z. R. He & Li Bing Zhang	KR006493	KR006436	KR006481
	*P. menglianensis* H. W. Li	KR006506	JN092510	JN092444
	*P. minor* Hemsl. Hook.	KR006515	JN092511	JN092445
	*P. nanchuanensis* sp. nov.	KR006489	KR006423	KR006472
	*P. nervosa* Craib	KR006523	JN092512	JN092446
	*P. oblata* Craib	KR006518	JN092513	GU350661
	*P. parryorum* C. E. C. Fisch.	KR006508	KR006426	KR006463
	*P. qinlingensis* W. T. Wang	KR006521	KR006419	KR006470
	*P. rosettifolia* C. Y. Wu & H. W. Li	KR006510	KR006428	KR006473
	*P. sericea* C. Y. Wu & H. W. Li	KR006500	JN092500	JN092434
	*P. shilinensis* Y. M. Shui & H. T. Zhao	KR006511	KR006434	KR006486
	*P. sinensis* Oliver	KR006522	JN092514	GU350660
	*P. xanthomaculata* G. Q. Gou & X. Y. Wang	KR006488	KR006421	KR006477
	*P. xingyiensis Y. G. Wei & F. Wen*	KR006502	KR006432	KR006485
	*P. yanshanensis* Z. J. Qiu & Y. Z. Wang	KR006499	JN092495	JN092429
	*P. barbata* Craib	KR006491	KR006424	KR006475
**Outgroups**	*Raphiocarpus begoniifolius* (Lévl) Burtt	KR006524	JN092515	GU350648
	*R. petelotii* (Pellegr) Burtt	KR006525	JN092516	JN092447

We performed comprehensive phylogenetic analyses using both maximum likelihood (ML) and Bayesian inference (BI) approaches. For the ML analysis, model selection was conducted using ModelFinder (Kalyaanamoorthy et al. 2017), which identified K3P+G4 as the optimal substitution model based on the Bayesian Information Criterion (BIC). This model incorporated unequal base frequencies (+F), the K3P substitution matrix, and among-site rate variation (+G4). Phylogenetic reconstruction was performed in IQ-TREE v2.3.6 (Minh et al. 2020), with branch support assessed via 1000 ultrafast bootstrap replicates (UFBoot ≥95% considered strong support; Hoang et al. 2018). For the BI analysis, the trimmed ITS sequence alignment was analyzed using MrBayes v3.2.7 (Ronquist and Huelsenbeck 2003). The analysis consisted of four independent MCMC runs of 10 million generations each, sampling every 1000 generations. After discarding the first 25% of samples as burn-in, posterior probabilities were calculated from the remaining trees to generate a majority-rule consensus tree. All phylogenetic trees were visualized and annotated using FigTree v1.4.4.

## ﻿Taxonomic treatment

### ﻿ Petrocosmea pengzhouensis

Taxon classificationPlantaeLamialesGesneriaceae

K.Huang & Z.X.Fu sp. nov.

A14A3E45-0B83-5E88-B2B5-F422EC170A56

urn:lsid:ipni.org:names:77368635-1

#### Type.

**Figure 1.**
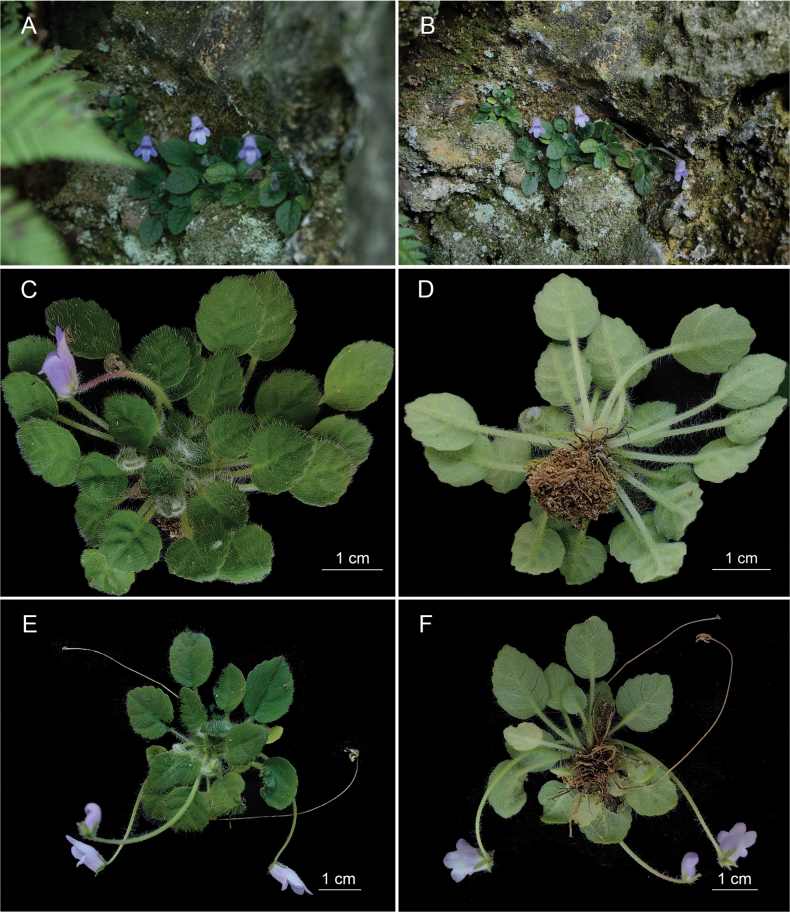
*Petrocosmea pengzhouensis* sp. nov. A, B. Habitat; C, E. Plant; D, F. Plant and roots (Photos A, B by KH and C–F by XZ).

**Figure 2.**
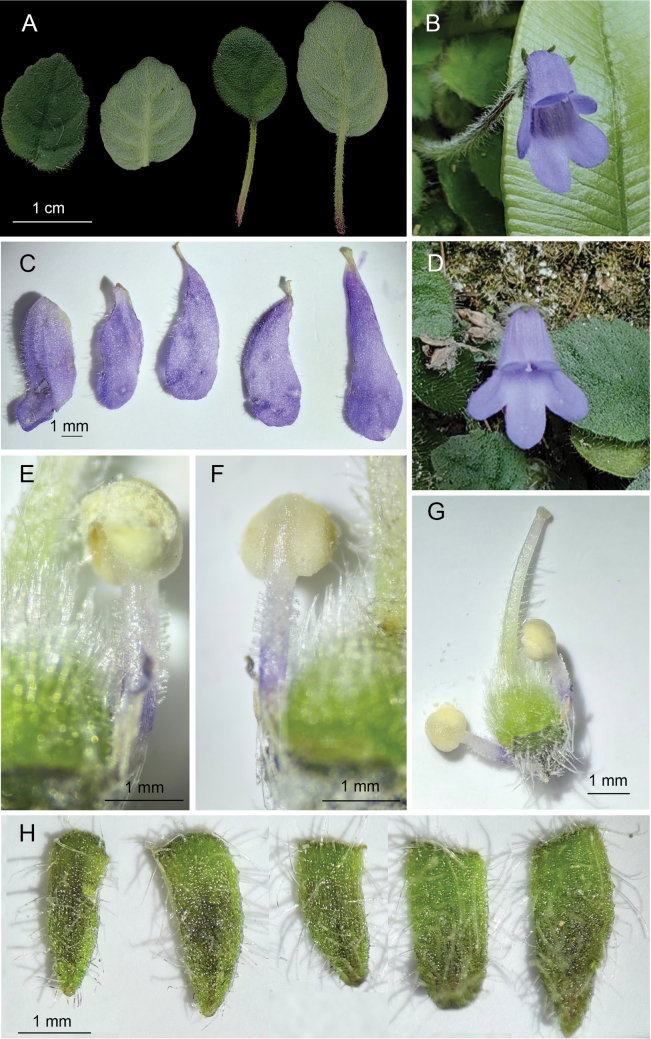
*Petrocosmea pengzhouensis* sp. nov. A. Leaves; B, D. Flowers; C. Dissected corollas; E, F. Stamens; G. Pistil and stamens; H. Sepals (Photos by XZ).

**Figure 3.**
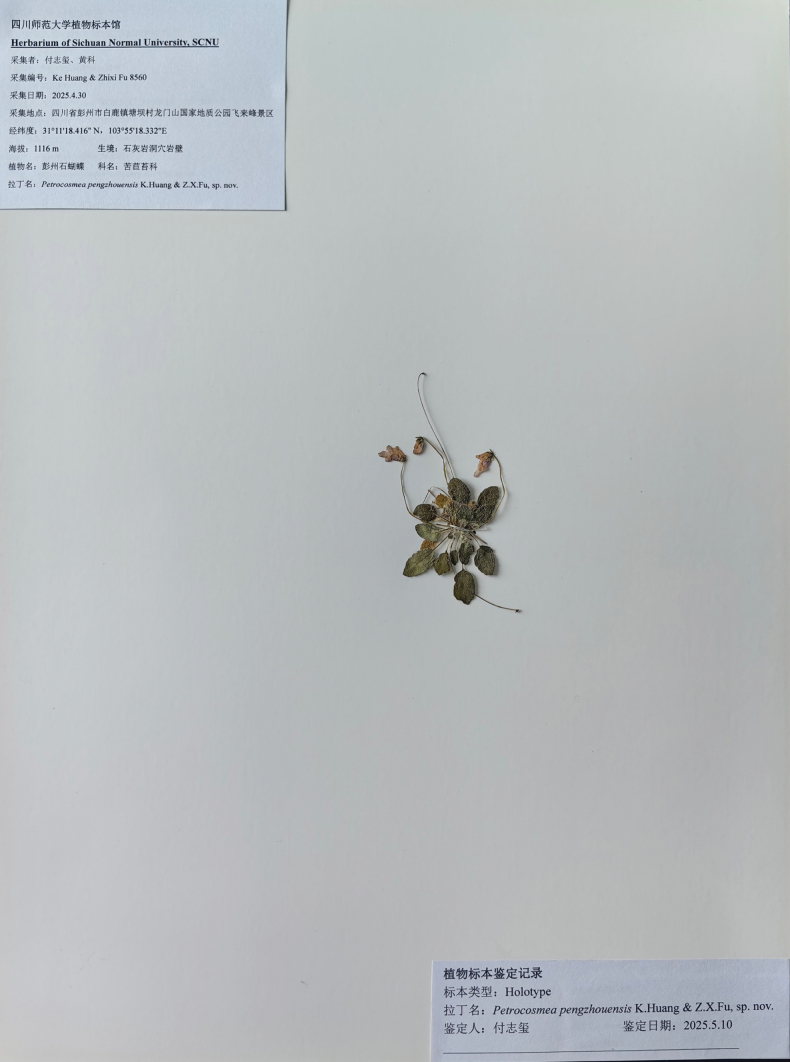
Holotype image of *Petrocosmea pengzhouensis* K.Huang & Z.X.Fu, sp. nov.

**Figure 4.**
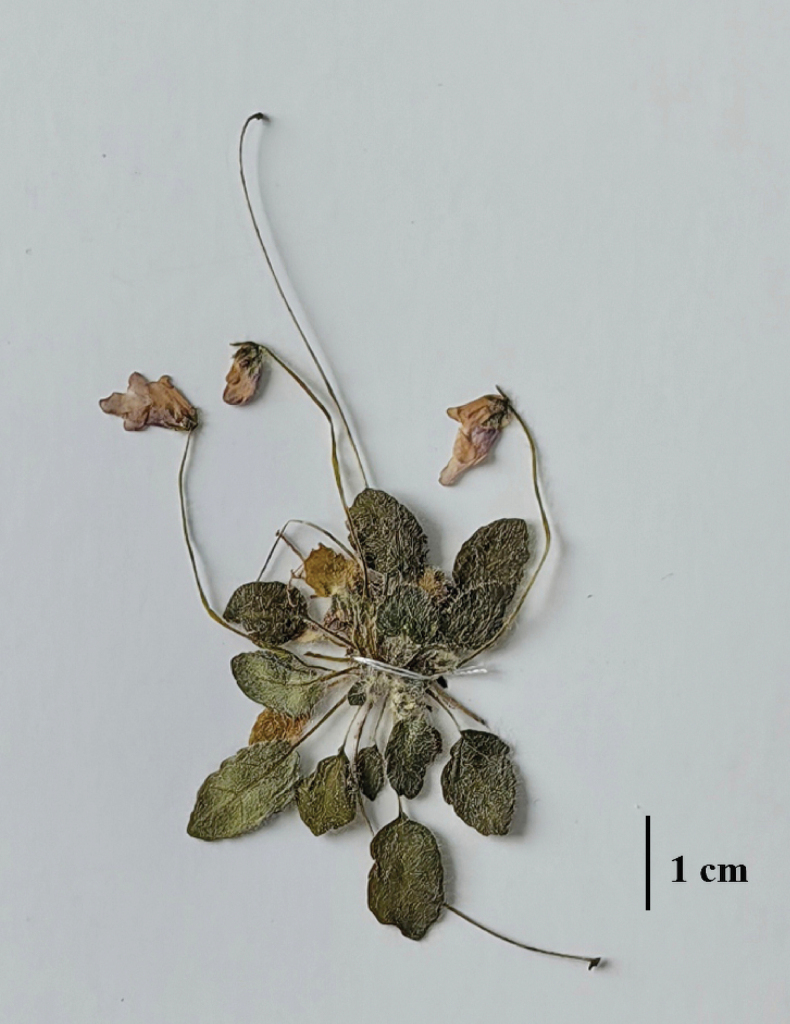
Close-up image of *Petrocosmea pengzhouensis* K.Huang & Z.X.Fu, sp. nov.

China • Sichuan Province, Chengdu City, Pengzhou, Feilaifeng Scenic Area of Sichuan Longmen Mountain National Geological Park, 31.31.193542, 103.917283, ca. 1116 m, 30 April 2025, *Ke Huang* & *Zhixi Fu* 8560 (holotype: SCNU!; isotype: SCNU!) (Figs 1–4).

#### Diagnosis.

In terms of traits such as being a rosette-forming herb, possessing a single-flowered cyme, and having a pale purple corolla, *P. pengzhouensis* most closely resembles *P. duclouxii* but differs primarily in the following characteristics: leaf shape (ovate to oblique-ovate vs. ovate to nearly orbicular), corolla throat (spotless vs. bearing two deep purple spots), and filament pubescence (translucent-puberulent vs. rust-brown puberulent) (Table 2).

**Table 2.** Morphological character comparison amongst *Petrocosmea pengzhouensis*, *P. hexiensis*, *P. duclouxii*, and *P. intraglabra*.

Character	*P. pengzhouensis*	*P. duclouxii*	*P. hexiensis*	*P. intraglabra*
**Leaf shape**	Ovate to oblique–ovate	Ovate to nearly orbicular	Ovate–rhombic to rhombic	Ovate to nearly orbicular or elliptic
Leaf base	Cordate (sometimes oblique)	Cordate	Truncate	Slightly oblique
Leaf number	13–26	5–25	20–60	7–20
Leaf blade indumentum	Adaxial: sparsely pilose; abaxial: densely appressed–pubescent	Both surfaces sparsely white–pilose	Both surfaces densely appressed–pubescent	Adaxial: pilose, abaxial: densely puberulent
Leaf margin	Repand–crenate	Entire or obscurely repand–crenate	Repand–crenate	Entire near base, crenate-dentate toward apex, apex acute to rounded
Leaf vein	3–4 per side, slightly convex or flat on abaxial side	2–3 per side, slightly convex or flat on abaxial side	3 per side, inconspicuous	2–3 per side, conspicuous
Bracts	Absent	Present	Present	Present
Cyme	1–5 cymes	1–7 cymes	6–15 cymes	1–5 cymes
The degree of upper lip cracking	Near the middle	Near base	Near base	Near base
Shape of upper lip lobe	Ovate	Semi–circular	Ovate	Narrowly ovate
The degree of lower lip cracking	Near the middle	Near the middle	Near the middle	Near the middle
Shape of lower lip lobe	Long–ovate	Round–ovate	Ovate	Obovate and the top is round
Corolla	Tube 4.3–6.4 mm long, throat blue-purple, unspotted	Tube 3–5.2 mm long, throat with 2 deep purple spots	Tube 4.5–5.5 mm long, throat with 2 deep purple spots	Tube 3–4 mm long, throat white, unspotted
**Stamen**	Filaments densely translucent-puberulent	Filaments rust–brown puberulent	Filaments sparsely pubescent	Filaments rust-brown puberulent
Pistil	Pistil 4.3–5.4 mm, style pubescent below middle upper part	Pistil ca. 6 mm, style sparsely puberulent below middle	Pistil ca. 7 mm, style sparsely pubescent below middle	Pistil 5.2–6 mm, style pilose near base

#### Description.

Perennial rosette-forming herbs. ***Roots*** fibrous, clustered. ***Leaves*** herbaceous, 13–26, basal, densely rosulate; inner leaves subsessile or with petioles to 2.2 cm long in outer leaves, densely pubescent and sparsely villous; leaf blade 0.4–1.8 cm long, 0.3–1.2 cm wide, ovate to oblique-ovate in outer leaves, apex obtuse to rounded, base cordate (sometimes oblique), margin crenate, adaxially sparsely villous, abaxially densely pubescent or sparsely villous; lateral veins 3–4 per side, slightly raised or flat abaxially. ***Cymes*** 1–5, each 1-flowered; peduncle 0.5–4.3 cm long, densely pubescent or sparsely villous. ***Bracts*** absent. ***Calyx*** zygomorphic, 5-parted to base; lobes narrowly lanceolate, 1.9–2.7 mm long, externally pubescent. ***Flowers*** pale purple, 8.7–13.8 mm long; corolla externally densely puberulent, internally glabrous; tube 4.3–6.4 mm long, throat 4–5 mm in diameter, base 2.5–3 mm in diameter, subcampanulate, throat blue-purple, unspotted; adaxial lip conspicuously shorter, 8–9 mm long, 2-lobed to the middle, lobes ovate, 2–2.3 mm long, 2–2.5 mm wide, slightly reflexed; abaxial lip 11–13.8 mm long, 3-lobed to the middle, lobes long-ovate, 2.6–3.0 mm long, 3.6–4.2 mm wide, slightly reflexed. ***Stamens*** 2, ca. 2.5 mm long; filaments adnate, ca. 1 mm above corolla base, 1.8–2.3 mm long, 0.3–0.5 mm in diameter, densely translucent-puberulent; anthers ovate, dehiscing longitudinally, ca. 1 mm long, 1.0–1.1 mm wide; thecae 2, parallel. ***Pistil*** 4.3–5.4 mm long; ovary densely villous, ovoid, ca. 1.3 mm long, 1.2–1.5 mm in diameter; style 3–4.1 mm long, 0.3 mm in diameter, sparsely pubescent below middle-upper portion, slightly geniculate (ca. 45°) above base; stigma capitate, ca. 0.4 mm in diameter. ***Capsule*** straight relative to pedicel, 2.9–3.5 mm long, 1.2–1.3 mm in diameter, oblong to ovoid, dehiscing loculicidally to base.

#### Phenology.

Flowering from April to May, fruiting in the wild is unknown; only capsules of the previous year were observed.

#### Etymology.

The new taxon is named after the type locality, Pengzhou, Chengdu City, China.

#### Vernacular name.

The Chinese name is “péng zhōu shí hú dié” (彭州石蝴蝶).

#### Distribution and habitat.

**Figure 5.**
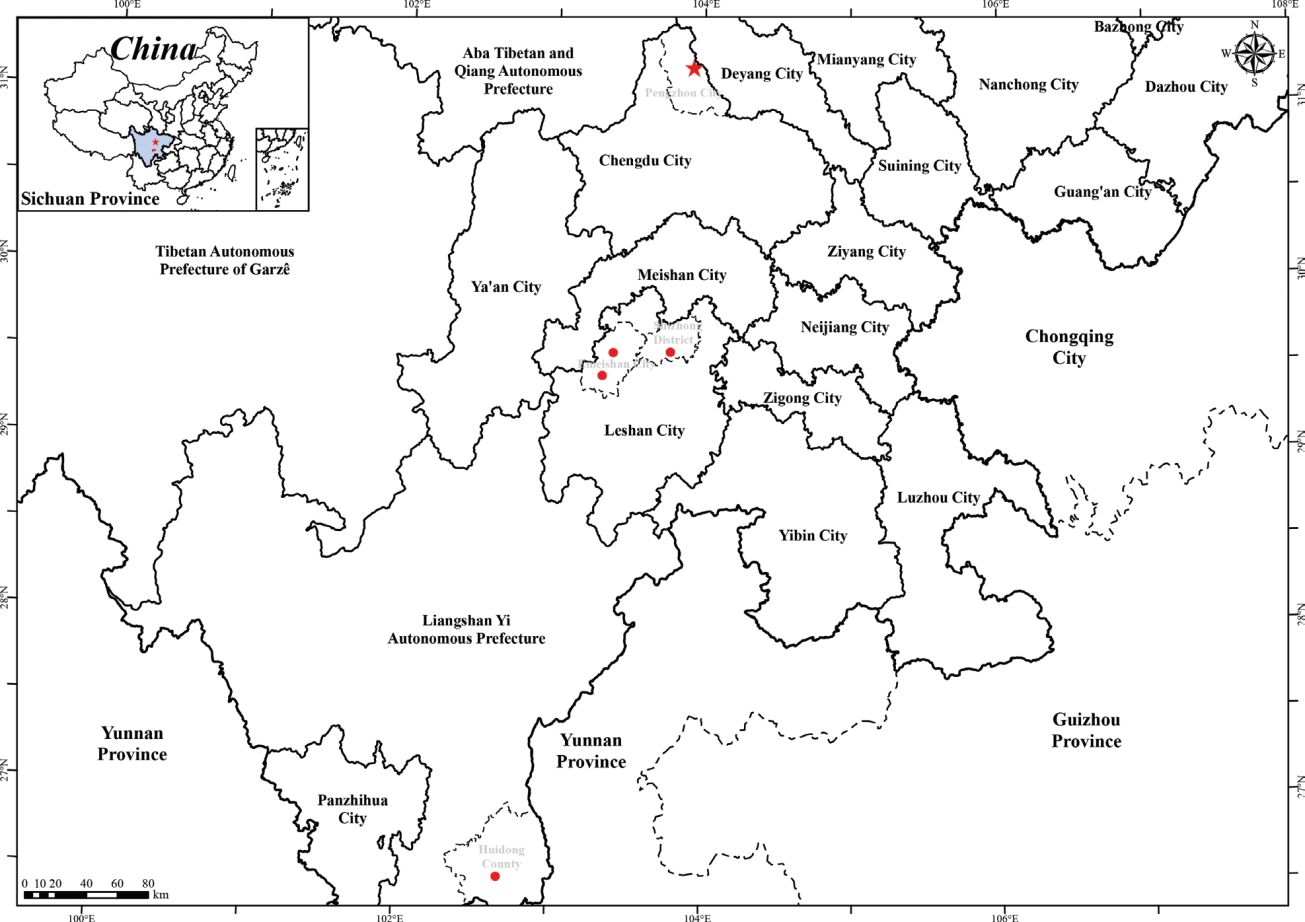
Distribution of *Petrocosmea pengzhouensis* and *P. duclouxii* in Sichuan Province, China (red star indicates *P. pengzhouensis*, red dots indicate *P. duclouxii*).

To date, only a single population has been found, exclusively in the Feilaifeng Scenic Area of the Longmen Mountain National Geological Park in Pengzhou, Chengdu City, Sichuan Province, China. *Petrocosmea pengzhouensis* was found growing on rocks in limestone caves at an elevation of ca. 1116 m (Fig. 5).

#### Conservation status and IUCN Red List category.

In the type locality, a population of approximately 100 mature individuals was discovered. This habitat is located within the local Feilaifeng Scenic Area, and it is highly likely that additional populations exist in the region. Before further investigations, this species should be assessed as “Data Deficient” (DD) according to the IUCN standards (IUCN 2024).

## ﻿Discussion

Morphologically, we observed that specimens collected from moist limestone cliffs in Pengzhou exhibited high similarity to *Petrocosmea duclouxii*, *P. hexiensis*, and *P. intraglabra*, particularly *P. duclouxii*. A detailed morphological comparison is provided in Table 2. *Petrocosmea duclouxii* and *P. pengzhouensis* are perennial rosette herbs with pubescent leaves on both surfaces and cymose inflorescences bearing a single flower, along with pale purple corollas. However, the newly discovered population also displayed distinct differences from the latter species. *P. duclouxii* is characterized by ovate to nearly orbicular leaves, two deep purple spots at the throat, and rust-brown puberulent filaments (Qiu et al. 2012). The newly discovered species from Pengzhou has ovate to oblique-ovate leaves, a spotless throat, and translucent-puberulent filaments. According to the Plant Photo Bank of China (PPBC, http://ppbc.iplant.cn/list21?latin=Petrocosmea%20duclouxii&pro=%E5%9B%9B%E5%B7%9D&sel=like), the Chinese Virtual Herbarium (CVH, https://www.cvh.ac.cn/spms/list.php?&taxonName=Petrocosmea%20duclouxii), and literature records (Li and Wang 2005; Qiu et al. 2012), the distribution of *P. duclouxii* specimens in Sichuan Province is primarily concentrated in Huidong County of the Liangshan Yi Autonomous Prefecture, as well as Mount Emei, Leshan Giant Buddha, and Longchi Sixigou in Leshan City. These locations exhibit significant geographical isolation from Pengzhou City.

**Figure 6.**
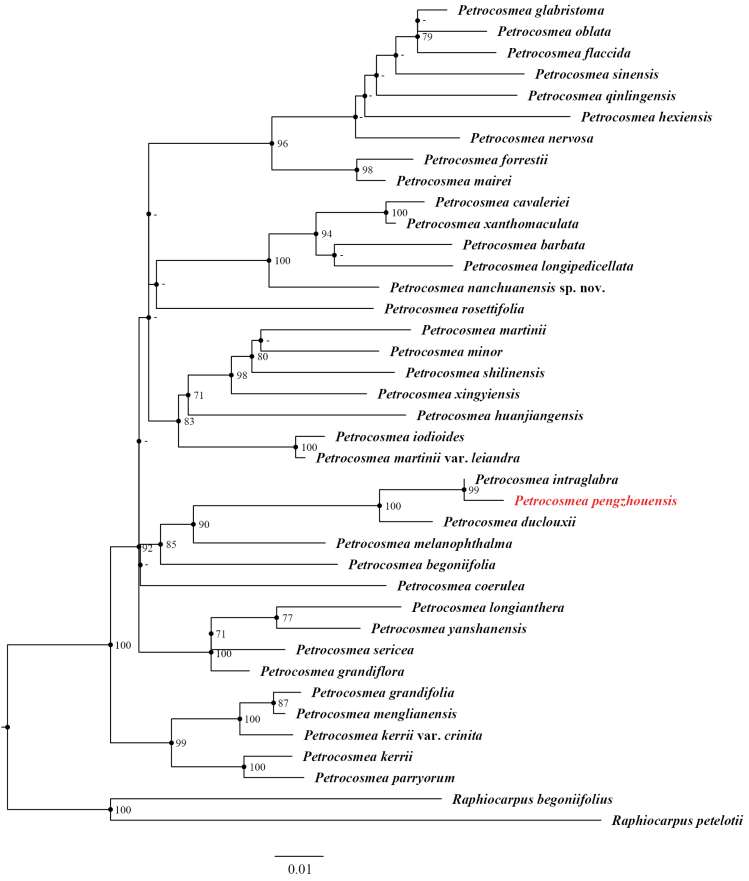
Phylogenetic reconstruction of 37 *Petrocosmea* species based on chloroplast regions (*rps*16 and *trn*T-L) and ITS sequences. *Petrocosmea pengzhouensis* is highlighted in red. Numbers at branches represent ML bootstrap (ML-BS) values; “-” indicates nodes with support values <70.

**Figure 7.**
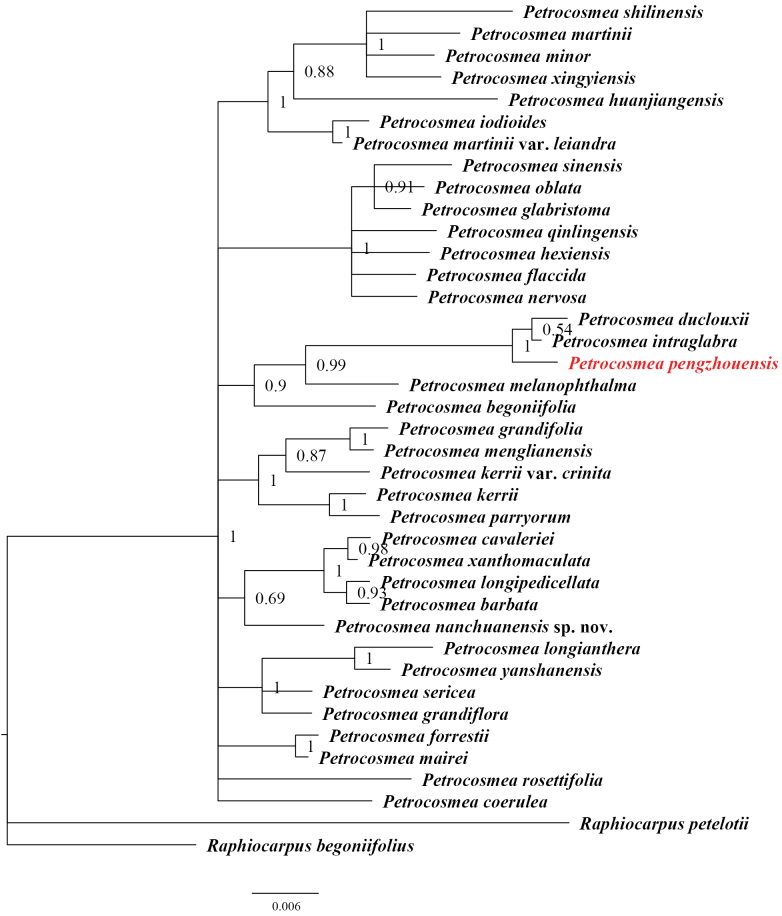
Phylogenetic reconstruction of 37 *Petrocosmea* species based on ITS sequences. The phylogenetic position of *Petrocosmea pengzhouensis* is marked in red font. The numbers on the branches represent Bayesian posterior probabilities (PP) values.

In this study, the ML tree (Fig. 6) revealed that the new species formed a highly supported clade (BS = 100) with *P. intraglabra* and *P. duclouxii*, and this clade was sister to *P. melanophthalma* (BS = 90). Similarly, in the BI tree (Fig. 7), the same three species clustered together (PP = 1) and were adjacent to *P. melanophthalma* (PP = 0.99). This consistent topological structure is supported by previous research findings (Qiu et al. 2015), confirming that both *P. intraglabra* and *P. duclouxii* are closely related to *P. pengzhouensis*.

**Figure 8.**
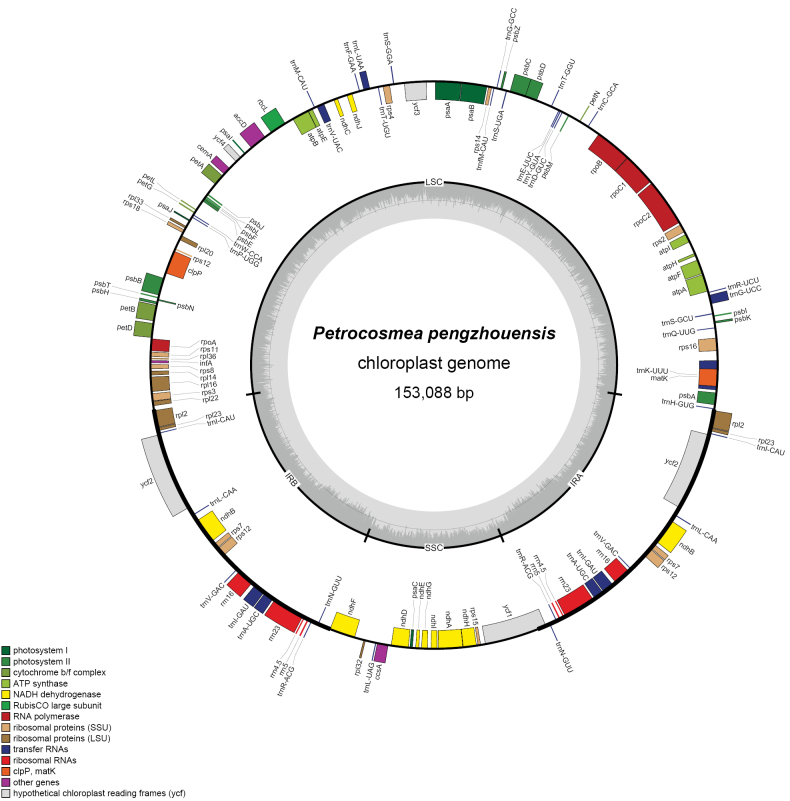
Gene map of the plastomes of *Petrocosmea pengzhouensis*.

Phylogenetic analysis also revealed a distant relationship between *P. pengzhouensis* and *P. duclouxii*, supported by distinct chloroplast gene counts and genomic features (Fig. 8, Table 3). The complete chloroplast genome of *P. pengzhouensis* (153,088 bp; total GC content: 35.57%) comprises an 82,885 bp large single-copy (LSC) region (GC: 35.57%), an 18,181 bp small single-copy (SSC) region (GC: 30.65%), and two 24,639 bp inverted repeat (IR) regions (GC: 43.01%). In comparison, *P. duclouxii* exhibits a slightly larger chloroplast genome (153,320 bp; total GC content: 37.60%) with an 84,322 bp LSC (GC: 35.60%), an 18,312 bp SSC (GC: 31.21%), and two 25,343 bp IRs (GC: 43.25%). Notably, both species display elevated GC content in their IR regions relative to single-copy regions, with *P. duclouxii* possessing marginally expanded IRs.

**Table 3.** Characteristics of complete chloroplast genomes of *P. pengzhouensis* and *P. duclouxii*.

Speciese	Genome size·(bp)	LSC size (bp)	IRa/Rb size·(bp)	SSC Size (bp)	Total. GC Content (%)	GC Content in·LSC (%)	GC Content in·IRa/IRb (%)	GC Content in·SSC (%)
*Petrocosmea pengzhouensis*	153, 088	82, 885	24, 639	18, 181	37.61	35.57	43.01	30.65
*Petrocosmea duclouxii*	153, 320	84, 322	25, 343	18, 312	37.60	35.60	43.25	31.21

In summary, morphologically, *P. pengzhouensis* closely resembles *P. duclouxii*, *P. hexiensis*, and *P. intraglabra*, particularly *P. duclouxii*, yet distinct differences remain. Molecular evidence supports *P. pengzhouensis* as a close relative of *P. duclouxii* and *P. intraglabra*. These findings confirm that *P. pengzhouensis* represents a new species within the genus *Petrocosmea*. Further molecular and morphological studies can help clarify the taxonomic status and evolutionary relationships of this species.

## Supplementary Material

XML Treatment for Petrocosmea pengzhouensis

## ﻿Additional information

### Use of AI

No use of AI was reported.

### Author ORCIDs

Xinyi Zheng https://orcid.org/0009-0003-2300-5525

Tianmeng Qu https://orcid.org/0009-0008-0185-5306

Xinyu Chen https://orcid.org/0009-0008-6347-2490

Yanru Zhang https://orcid.org/0009-0009-0450-2001

Yujie Wang https://orcid.org/0009-0004-0732-2827

Bo Li https://orcid.org/0000-0002-5131-8639

Ke Huang https://orcid.org/0009-0006-2057-5452

Zhixi Fu https://orcid.org/0000-0002-2789-6287

### Data availability

All of the data that support the findings of this study are available in the main text.
